# Supplementary figures and images for: Dual silencing of lipophagy and lipolysis in Rhodnius prolixus induces lipid droplet remodeling without TAG accumulation in the fat body
Source: PLoS One. 2025 Nov 7;20(11):e0336411. doi: 10.1371/journal.pone.0336411 (PMC12594430; doi:10.1371/journal.pone.0336411)

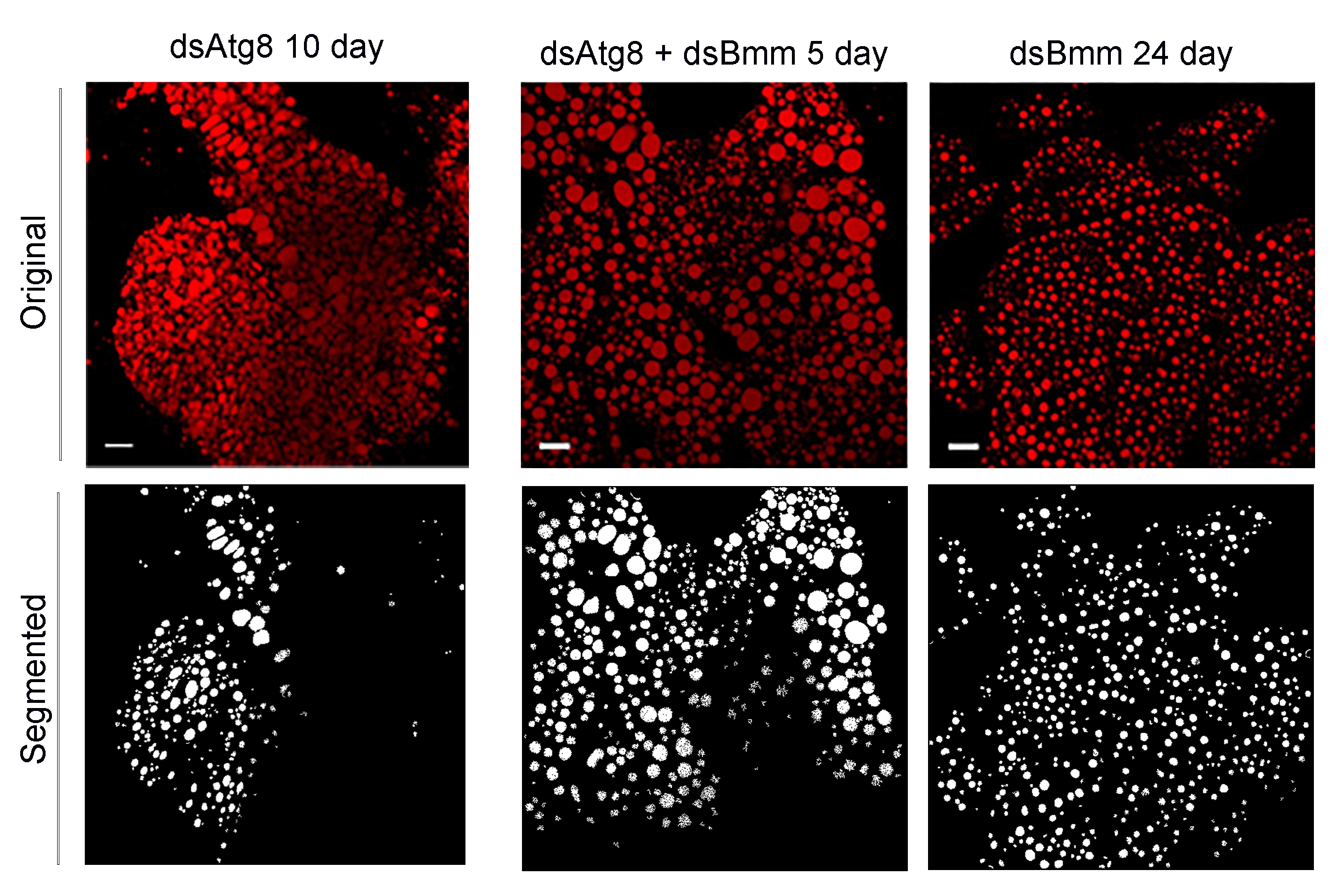

Supplement: S1 Fig — Examples of DAIME-based segmentation are shown for (A) day 10 dsAtg8 and (B–C) two additional representative experimental conditions. Lipid droplets were stained with Nile Red and segmented using the Marr-Hildreth edge detection algorithm with watershed set to 12% and exclusion of dark regions. No manual refinement was required. These images illustrate the basis for the lipid droplet diameter quantification presented in the manuscript. (TIF) [file pone.0336411.s001.tif]

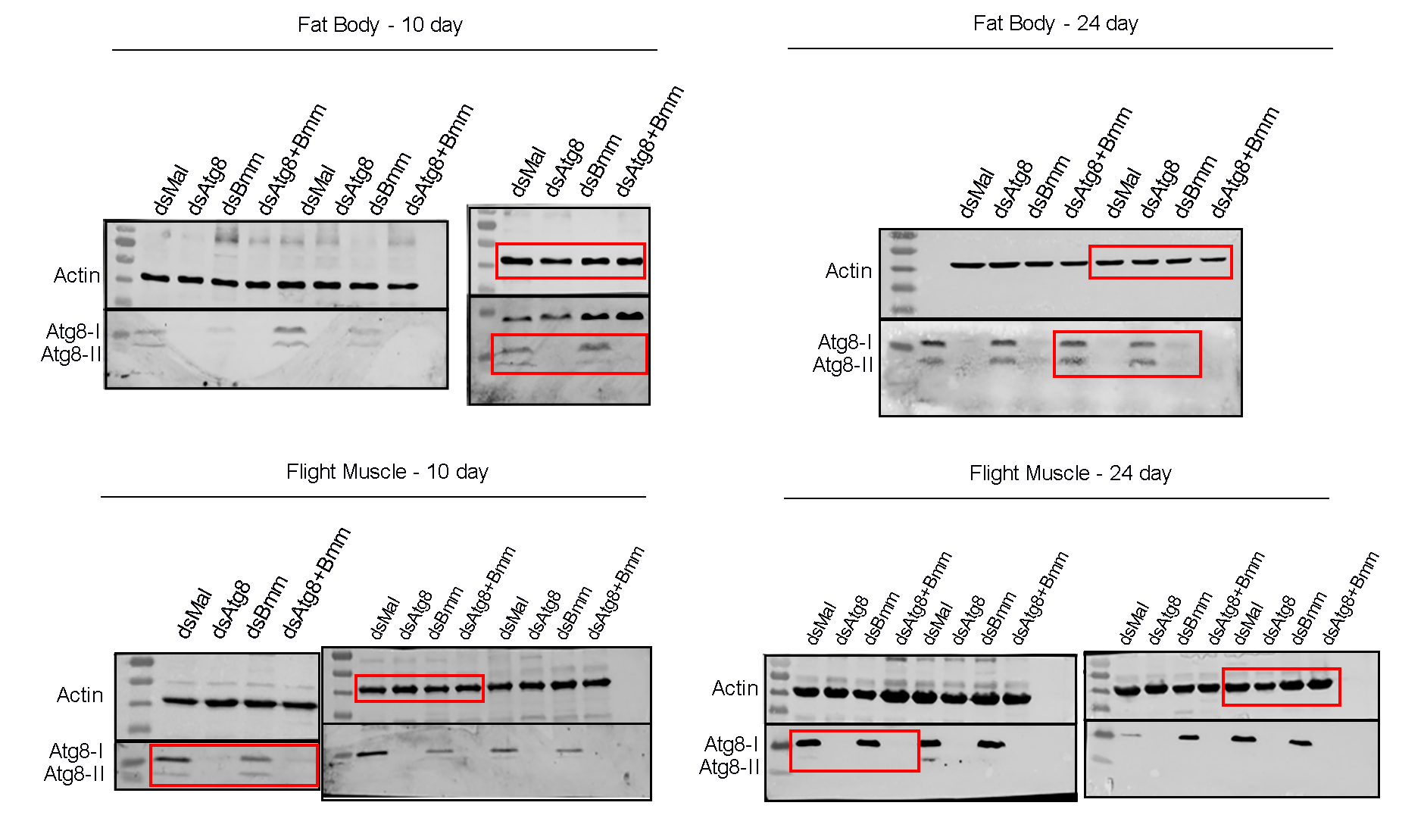

Supplement: S2 Fig — Uncropped images of the original blots are shown to document the full experimental results. The specific regions used in the main figures are indicated in red. (TIF) [file pone.0336411.s002.tif]

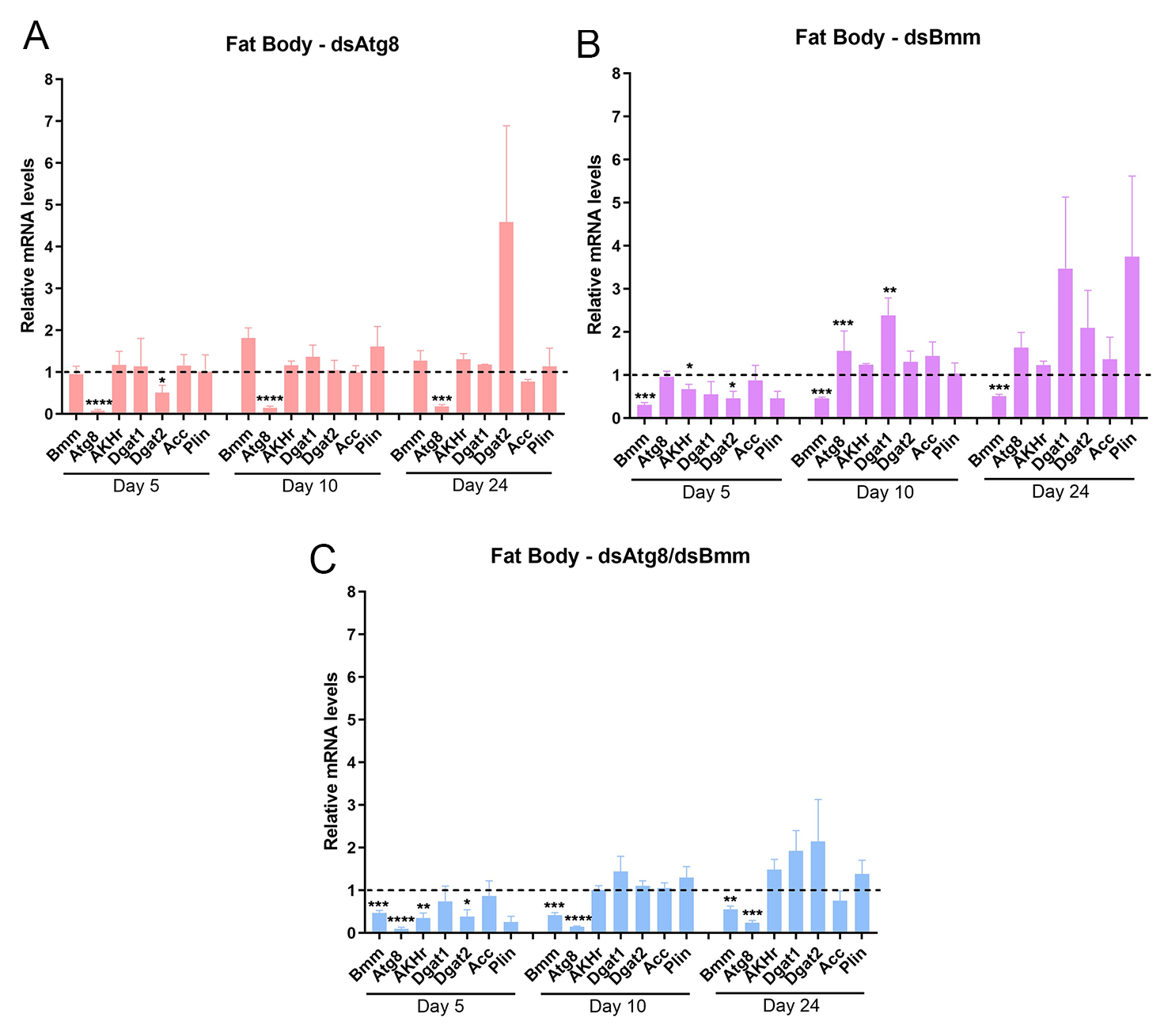

Supplement: S3 Fig — Adult females (18 days after a blood meal) were injected with 1 μg of dsRNA for RpAtg8, RpBmm, RpAtg8 + RpBmm, or Mal (control), fed three days later, and dissected either five or ten days after feeding (first protocol) or injected on the tenth day after feeding and dissected 14 days after injection (second protocol). Gene expression levels in the fat body were determined by qPCR using specific primers designed to target different genes. Rp18S amplification was used as an endogenous control. Gene expression levels are relative to each control value (dashed line). The graphs show mean ± SEM of 5 independent determinations, n = 5. *p < 0.05, **p < 0.01, ***p < 0.001, ****p < 0.0001, when compared by Student’s t-test. Akhr, adipokinetic hormone receptor; ACC, acetyl-CoA carboxylase; DGAT1, diacylglycerol acyltransferase 1; DGAT2, diacylglycerol acyltransferase 2; Plin, perilipin. (TIF) [file pone.0336411.s003.tif]

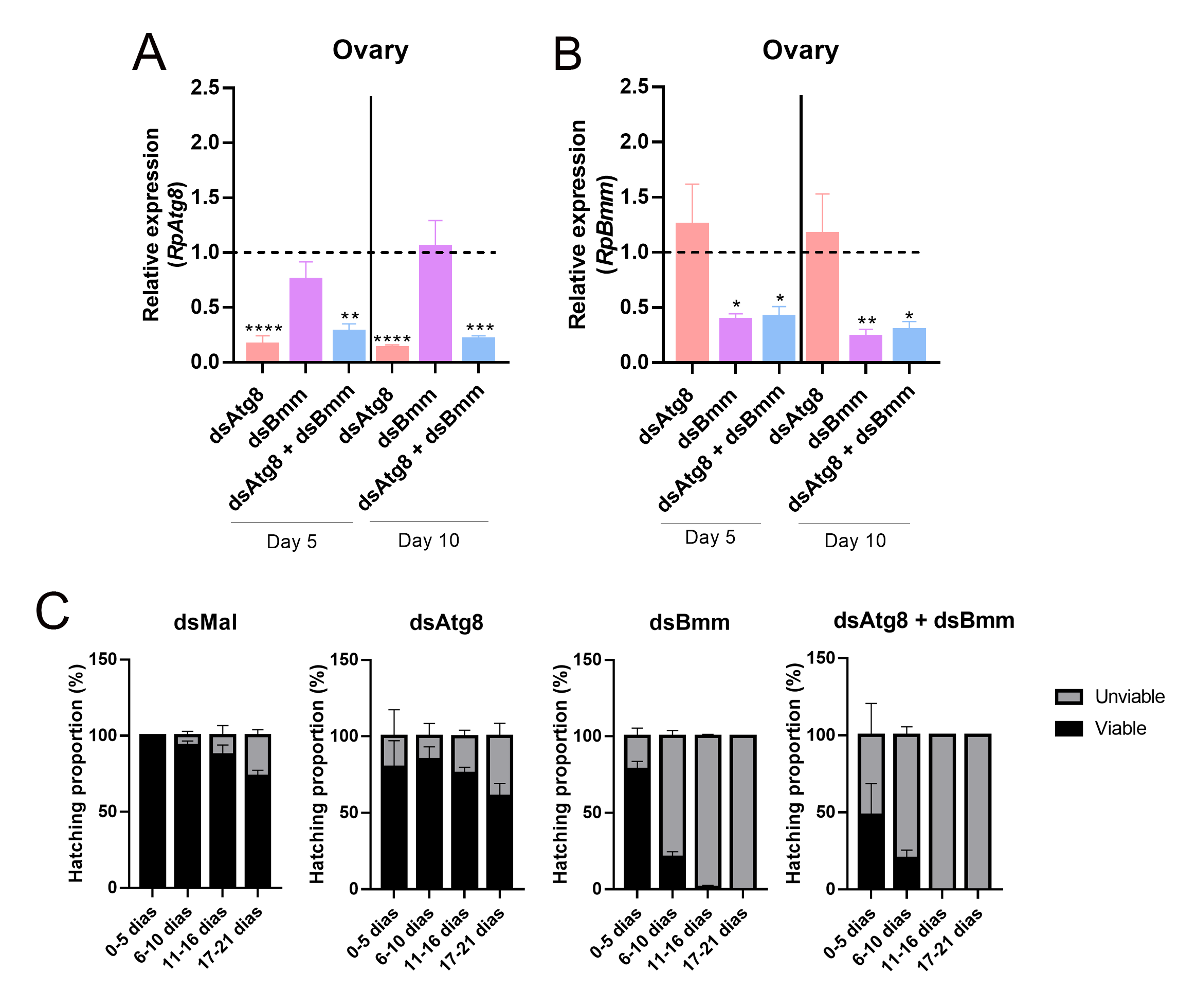

Supplement: S4 Fig — Adult females (18 days after a blood meal) were injected with 1 μg of dsRNA for RpAtg8, RpBmm, RpAtg8 + RpBmm, or Mal (control), fed three days later, and dissected five or ten days after feeding (protocol 1). (A–B) Quantification of RpAtg8 and RpBmm mRNA in the ovary. mRNA levels were determined by qPCR, using Rp18S expression as a reference gene. (C) Hatching proportions of eggs laid on different days after feeding. Graphs show mean ± SEM (n = 4). *p < 0.05, **p < 0.01, ***p < 0.001, ****p < 0.0001, when compared by one-way ANOVA followed by Tukey’s post-test. (TIF) [file pone.0336411.s004.tif]
