# Supplementary material for: Dual silencing of lipophagy and lipolysis in Rhodnius prolixus induces lipid droplet remodeling without TAG accumulation in the fat body
Source: PLoS One. 2025 Nov 7;20(11):e0336411. doi: 10.1371/journal.pone.0336411 (PMC12594430; doi:10.1371/journal.pone.0336411)
Supplement: S1 Table — Sequences of primers used in qPCR experiments and dsRNA synthesis. Sequences were obtained from VectorBase (https://www.vectorbase.org/) or previous works and synthesized by Macrogen or IDT technologies. (DOCX) [file pone.0336411.s005.docx]

**Table S1: Genes and primers list.** Sequences of primers used in qPCR experiments and dsRNA synthesis. Sequences were obtained from VectorBase (https://www.vectorbase.org/) or previous works and synthesized by Macrogen or IDT technologies.

| **GENE** | **VECTORBASE** | **PRIMER SEQUENCE (5’-3’)** | **Reference** |
| --- | --- | --- | --- |
| **18s** | RPRC017412 | FOR: TCGGCCAACAAAAGTACACA  REV: TGTCGGTGTAACTGGCATGT | (Majerowicz et al., 2011) |
| **Atg8 (qPCR)** | RPRC014434 | FOR: GAACAATGTAATCCCACCGACAA  REV: CCATAGACATTTTCATCACTATACG | (Pereira et al., 2020) |
| **Atg8 (dsRNA)** |  | FOR: TAATACGACTCACTATAGGGTACTATGAAGTTTCAATATAAAGAAGAGC  REV: TAATACGACTCACTATAGGGTACTATCTTCTTCATGATGTTCCTGAT | (Pereira et al., 2020) |
| **Bmm (qPCR)** | RPRC002097 | FOR: ATCTTCTTCATGATGTTCCTGAT  REV: GGGCATATATCGGTTTCACC | (Arêdes et al., 2024) |
| **Bmm (dsRNA)** |  | FOR: TAATACGACTCACTATAGGGCCGTGAAGTAGCGTCGGAAT  REV: TAATACGACTCACTATAGGGAGCAATAAAGCACCCAGGCT | (Arêdes et al., 2024) |
| **Cpt1 (qPCR)** | RPRC005639 | FOR: AAACACCACATGGCCAAACT  REV: GAAACGCCGTATCCATCATC | (De Paula et al., 2023) |
| **AKHr (qPCR)** | - | FOR: TTCTATTCGCATGCACCAAC  REV: ACTAGTGCGCGAGTTGTTTG | (Alves-Bezerra et al., 2016) |
| **Acc (qPCR)** | RPRC013987 | FOR: TGGGCTGGAACCGTAGTTGCG  REV: TGCGGGATCGGCTGGAAGTTGT | (Moraes et al., 2022) |
| **Dgat1 (qPCR)** | RPRC003681 | FOR: TCACAACCGGATAAACCTTG  REV: TGAGAGCCAGTCAACACTAT | (Arêdes et al., 2024) |
| **Dgat2 (qPCR)** | RPRC002968 | FOR: CCGGCTCACTTACTTACAAC  REV: CGATTGGCTTTCCAACTACA | (Arêdes et al., 2024) |
| **Plin1 (qPCR)** | RPRC013800 | FOR: TCAAGACCTGCCGAAACTGA  REV: TGTTGATGCTCCTTCTTGCG | Present study |
