# Supplementary material for: Dual silencing of lipophagy and lipolysis in Rhodnius prolixus induces lipid droplet remodeling without TAG accumulation in the fat body
Source: PLoS One. 2025 Nov 7;20(11):e0336411. doi: 10.1371/journal.pone.0336411 (PMC12594430; doi:10.1371/journal.pone.0336411)
Supplement: Attachment — Submitted filename: Santos-Araujo et al., 2025 authors answers_R2.docx [file pone.0336411.s008.docx]

**Response to Reviewers - PONE-D-25-44176 - R2**

**Reviewer #1**

Reviewer #1: The authors addressed most of my questions satisfactorily, except for question 3. The images in Fig. S1 R1 do not show the segmentation output (binary; black-and-white); instead, they are still grayscale images and apparently do not represent the segmentation data produced by the software. To access the segmentation, one must extract it from the segmentation data layer (this is a fast, simple process in DAIME 3: Import the data in DAIME. After segmentation, select the imported original image. Right-click and choose “Extract object layer”. The segmentation layer appears below. Then right-click the segmentation layer and select “Show in Visualizer”. In the Visualizer, click the camera icon to take a snapshot and save it.
The segmented images are the basis for the quantification of the LDs (diameters, histograms). Based on these data, one can evaluate the efficiency of LD detection and, particularly, how closely associated and more diffuse LDs were separated. Since reliable image-based LD detection can be challenging and is used in different fields, demonstrating the efficiency of DAIME for single-LD detection/separation in such images would be valuable. Furthermore, these images serve as a control for the quantification process and related outcome. Please provide the segmentation data.

**Authors’ response:** We sincerely apologize for the confusion caused in our previous revision. In our first response, we mistakenly submitted grayscale (black-and-white) images instead of the actual segmented layer images extracted from the DAIME software. We would like to emphasize that this was purely a submission error and not an intentional omission or withholding of data. We fully acknowledge the reviewer’s concern and understand the importance of presenting the correct segmentation outputs that correspond to the original images, as these are essential for validating the accuracy of LD quantification.

We have now corrected this oversight and are providing, for your evaluation, the segmented layer images corresponding to four representative examples from each of the time points analyzed in *Atg8*-silenced fat bodies. These images were properly extracted from the segmentation layer in DAIME and directly represent the datasets used for LD quantification.

Figure S1 was revised to show the actual segmentation layers of three representative images.

We thank the reviewer for the careful and constructive revision.
